# Motor neuron preservation and decrease of *in vivo* TDP-43 phosphorylation by protein CK-1δ kinase inhibitor treatment

**DOI:** 10.1038/s41598-020-61265-y

**Published:** 2020-03-10

**Authors:** Loreto Martínez-González, Carmen Rodríguez-Cueto, Diego Cabezudo, Fernando Bartolomé, Pol Andrés-Benito, Isidro Ferrer, Carmen Gil, Ángeles Martín-Requero, Javier Fernández-Ruiz, Ana Martínez, Eva de Lago

**Affiliations:** 1 Centro de Investigaciones Biológicas Margarita Salas-CSIC, Ramiro de Maeztu 9, 28040 Madrid, Spain; 2 Instituto Universitario de Investigación en Neuroquímica, Departamento de Bioquímica y Biología Molecular, Facultad de Medicina, Universidad Complutense, Madrid, Spain; 3 Instituto Ramón y Cajal de Investigación Sanitaria (IRYCIS), Madrid, Spain; 4 Centro de Investigación Biomédica en Red de Enfermedades Neurodegenerativas (CIBERNED), Madrid, Spain; 5 Instituto de Investigación Sanitaria Hospital 12 de Octubre (imas12), 28041 Madrid, Spain; 6 Department of Pathology and Experimental Therapeutics, University of Barcelona, Hospitalet de Llobregat, Barcelona, Spain; 7 Bellvitge University Hospital, IDIBELL (Bellvitge Biomedical Research Centre), Hospitalet de Llobregat, Barcelona, Spain

**Keywords:** Amyotrophic lateral sclerosis, Molecular neuroscience, Molecular medicine

## Abstract

Pathogenesis of amyotrophic lateral sclerosis (ALS), a devastating disease where no treatment exists, involves the compartmentalization of the nuclear protein TDP-43 (TAR DNA-binding protein 43) in the cytoplasm which is promoted by its aberrant phosphorylation and others posttranslational modifications. Recently, it was reported that CK-1δ (protein casein kinase-1δ) is able to phosphorylate TDP-43. Here, the preclinical efficacy of a benzothiazole-based CK-1δ inhibitor IGS-2.7, both in a TDP-43 (A315T) transgenic mouse and in a human cell-based model of ALS, is shown. Treatment with IGS-2.7 produces a significant preservation of motor neurons in the anterior horn at lumbar level, a decrease in both astroglial and microglial reactivity in this area, and in TDP-43 phosphorylation in spinal cord samples. Furthermore, the recovery of TDP-43 homeostasis (phosphorylation and localization) in a human-based cell model from ALS patients after treatment with IGS-2.7 is also reported. Moreover, we have shown a trend to increase in CK-1δ mRNA in spinal cord and significantly in frontal cortex of sALS cases. All these data show for the first time the *in vivo* modulation of TDP-43 toxicity by CK-1δ inhibition with IGS-2.7, which may explain the benefits in the preservation of spinal motor neurons and point to the relevance of CK-1δ inhibitors in a future disease-modifying treatment for ALS.

## Introduction

Amyotrophic Lateral Sclerosis (ALS) is a fatal progressive neurodegenerative disease, which results in the destruction of upper and/or lower motor neurons in the brain and spinal cord. It usually affects people between 40 and 60-year-old and the average survival from onset to death is 3–4 years^1^. Despite the severity of the disease and the high health care and social costs, no cure or viable long-term effective treatment has been identified, with only two therapeutic agents (riluzole and edaravone) already approved by FDA (Food and Drug Administration) but having limited efficacy or serving only for specific groups of patients^2^.

In 2006, the trans-activating response region DNA binding protein of 43 KDa, known as TDP-43, was identified as one of the main hallmarks of sporadic and familial ALS, showing accumulations in the cytoplasm of cortical and spinal motor neurons^3^. Mutations in this protein have been associated with cases of ALS (accounting for approximately 5% of genetic cases), but also have been associated with tau protein-independent cases of frontotemporal dementia (FTD), generating the idea of a pathological spectrum between ALS and FTD based on alterations in TDP-43 and other related proteins^4^. Today, it is recognized that TDP-43 proteinopathy, characterized by hyperphosphorylation, truncation, ubiquitination, and/or nuclear depletion in neurons, is the prominent and common pathological feature of sporadic and familiar ALS^1^. Furthermore, it is present in rare diseases such as Perry syndrome or Alexander disease, but also in the prevalent Alzheimer’s disease (AD) and recently in the limbic-predominant age-related TDP-43 encephalopathy (LATE)^2,3^. In addition, TDP-43 pathology is a secondary feature of several other neurodegenerative disorders, including Parkinson’s disease (PD), and Huntington’s disease (HD), where its presence may aggravate the primary existing proteinopathy^4^.

The study of the post-translational regulation of TDP-43 has placed the phosphorylation of this protein at specific residues, which is dependent on certain protein kinases, as a key event for regulating its cellular activities and also for its dysregulation associated with pathological conditions^5,6^. In fact, phospho-TDP-43 is distributed in brain areas of FTD and ALS patients^5^. Different kinases have been recently involved in TDP-43 phosphorylation. Among these, protein casein kinase-1 (CK-1)^7^, tau tubulin kinase 1 (TTBK1)^6^ and cell division cycle kinase 7 (CDC7)^7^ are the best characterized.

CK-1 was the first kinase identified to phosphorylate TDP-43 *in vivo* in more than 29 different sites, being 18 of them located in the C-terminal glycine-rich region^7^. Moreover, different stress signaling cause CK-1-dependent phosphorylation of TDP-43 triggering its cytosolic mislocalization and accumulation^8,9^. Furthermore, TDP-43 binds directly to and regulates the expression of CK-1ε mRNA^10^.

CK-1 is a highly conserved Ser/Thr kinase, constitutively active and ubiquitously expressed in eukaryotic organisms, with different human isoforms characterized (α, ɣ_1–3_,δ and ε)^8^. It is tightly regulated in cells because its role in crucial cellular processes. However, its dysregulation leads to different pathologies including cancer and neurodegenerative diseases^11^. Recently, inhibition of CK-1, mainly the δ and ε isoforms, has been proposed as a potential treatment for different neurodegenerative diseases including ALS, FTD^9^ and Alzheimer’s disease^12^.

We have discovered and synthetized new families of potent CK-1δ and dual CK-1δ/ε inhibitors with high selectivity score over other kinases based on the modification of the benzothiazole scaffold^10^. They have been proven to decreased TDP-43 phosphorylation both in cellular models and *in vivo* using a *Drosophila* transgenic model^11^. Moreover, two of these candidates, named as IGS-2.7 and IGS-2.37, have also shown a decrease on TDP-43 phosphorylation and nuclear localization using a cell-based model of human lymphoblast from FTD patients carrying a *progranulin (GRN)* mutation^12^. Therefore, our working hypothesis is that inhibitors of CK-1δ able to modulate TDP-43 proteinopathy *in vivo*, may be a good therapeutic strategy for the severe ALS. We have treated the TDP-43 (A315T) transgenic mice, one of the first experimental models of ALS based on mutations in TDP-43 protein^13^, with the brain permeable CK-1 inhibitor IGS-2.7. Our results show, for the first time, an *in vivo* decrease of TDP-43 phosphorylation, together with motor neuron survival and decrease of both astroglial and microglial reactivity. Furthermore, using lymphoblasts from sALS patients we have showed the recovery of TDP-43 homeostasis (phosphorylation and localization) after the treatment with the CK-1δ inhibitor. Moreover, an increase of *CSKN1D* mRNA in spinal cord and frontal cortex from patients of sporadic ALS (sALS) are also shown. All these data may serve as a solid proof of concept for the potential therapy of ALS with CK-1δ or dual CK-1δ/ε inhibitors showing a promising neuroprotective effect.

## Results

### Treatment with IGS-2.7, a CK-1δ inhibitor, in TDP-43 (A315T) transgenic mice

**Figure 1:**
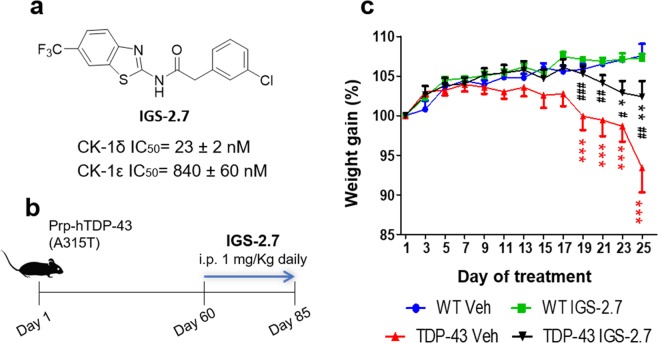
*In vivo* experiment with IGS-2.7. (**a**) Chemical structure of IGS-2.7 and its IC_50_ values on CK-1. (**b**) Experimental design of i.p. administration of the compound IGS-2.7 (**c**) Effects of IGS-2.7 treatment on body weight gain in TDP-43 and wild-type mice. Values are expressed as means ± SEM; N ≥ 8 animals in each group. Data were assessed by repeat measures two-way ANOVA followed by the Bonferroni test (*p < 0.05, **p < 0.01, ***p < 0.001 vs. WT-Veh group; ^#^p < 0.05, ^##^p < 0.01, ^###^p < 0.001 vs. TDP-43-Veh).

The first objective of our study was to explore the effects of a chronic treatment with the compound IGS-2.7 in the TDP-43 (A315T) transgenic mouse model. To this end, TDP-43 (A315T) transgenic mice and wild-type animals were daily treated with the inhibitor IGS-2.7 or vehicle from the age of 65 days up to 90 days. First, we observed the expected progressive decrease in animal weight in TDP-43 (A315T) transgenic mice, which was partially delayed after the treatment with IGS-2.7. This effect of IGS-2.7 in transgenic TDP-43 (A315T) mice weight was statistically significant from the day 19 of treatment (Fig. 1), although they resulted to be statistically different compared to wild-type animals at the last two time-points analysed (23 and 25 days of treatment) (Fig. 1).

**Figure 2:**
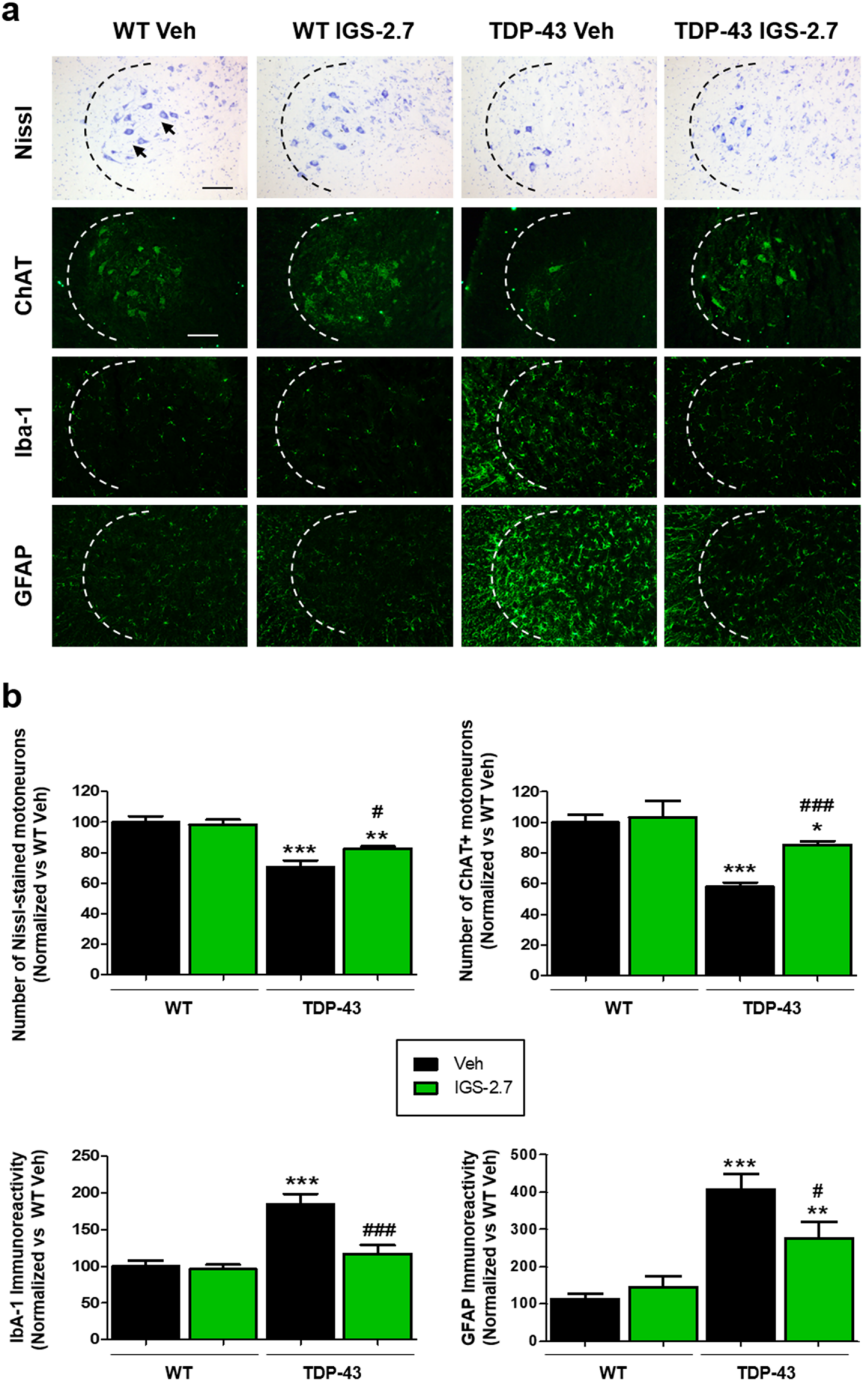
Photomicrographs of histological samples in the anterior horn of the spinal cord of TDP-43 mice and wild-type controls. (**a**) Representative images of Nissl staining of motor neurons (black arrows), ChAT, Iba-1 and GFAP immunostained sections (**b**) Quantification of the different markers. Total average number of motor neurons (Nissl, ChAT+), microglia (Iba-1+) and astrocytes (GFAP+) is shown. Data are expressed as a percentage over the WT-Veh group. Values are expressed as means ± SEM, N ≥ 6 in each group. Data were assessed by one-way ANOVA followed by the Bonferroni test (*p < 0.05, **p < 0.01, ***p < 0.001 vs. WT-Veh group; ^#^p < 0.05, ^##^p < 0.01, ^###^p < 0.001 vs. TDP-43-Veh). Scale bars = 200 μm.

We next investigated the effect of IGS-2.7 against the spinal motor neuron degeneration typical of TDP-43 (A315T) transgenic mice. A reduction in the number of motor neurons was evident in the anterior horn at lumbar level by using both Nissl staining and choline acetyl transferase (ChAT) immunohistochemistry (Fig. 2). The treatment with IGS-2.7 rescued the loss of both Nissl and ChAT-positive cells in TDP-43 (A315T) transgenic mice (Fig. 2), stressing an important neuroprotective effect in this experimental model of ALS.

Glial reactivity and associated inflammatory events have been claimed as important pathogenic players in ALS neurodegeneration^14^. We investigated the effect of this treatment on astroglial and microglial reactivity labelled by GFAP and Iba-1 immunostaining, respectively. TDP-43 transgenic mice showed statistically significant elevations in both microglial and astrocyte immunoreactivities compared to wild-type animals, whereas the treatment with IGS-2.7 completely blocked the elevated immunoreactivity of microglial cells and partially reduced the immunoreactivity of astrocytes compared to the controls (Fig. 2).

**Figure 3:**
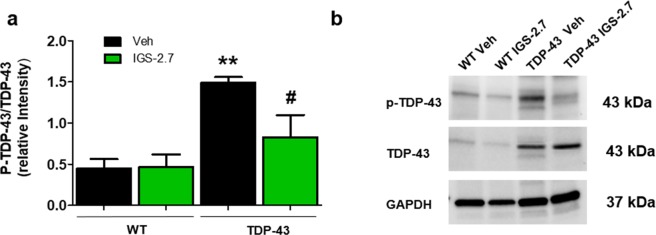
Effects of IGS-2.7 on phosphorylation of TDP-43 in the spinal cord of wild-type and transgenic TDP-43 mice. (**a**) Spinal cord lysates from wild-type and transgenic TDP-43 mice were used to determine the levels of pTDP-43 and TDP-43. (**b**) Representative immunoblots are shown. Densitometric analyses represent the ratio of pTDP-43/total TDP-43 and are the mean ± SEM of 8 observations per group. Data were assessed by one-way ANOVA followed by the Bonferroni test (*p < 0.05 WT-Veh group; ^#^p < 0.05 vs. TDP-43-Veh).

It is believed that abnormal phosphorylation of TDP-43 is a critical step of FTD-TDP and ALS^15^. On these grounds, we investigated whether our CK-1δ inhibitor IGS-2.7 chronic treatment could prevent the enhanced TDP-43 phosphorylation *in vivo* in these transgenic mice. To this end, we analysed the levels of both phosphorylated and total TDP-43 in spinal cord samples of TDP-43 (A315T) transgenic mice and wild-type animals by immunoblotting with specific antibodies. We observed a robust increase in the ratio of pTDP-43/TDP-43 in TDP-43 (A315T) transgenic mice compared with wild-type animals (Fig. 3), which was completely reversed, at values similar to wild-type animals, by the treatment with IGS-2.7 (Fig. 3).

Lastly, it is important to remark that the positive effects of IGS-2.7 in maintaining animal weight, preserving spinal motor neurons and limiting glial reactivity were exclusively found in TDP-43 transgenic mice treated with the inhibitor causing no effects when is administered to wild-type animals (Figs. 1–3).

### Effect of IGS-2.7 on TDP-43 phosphorylation of lymphoblasts from control and sporadic ALS individuals

**Figure 4:**
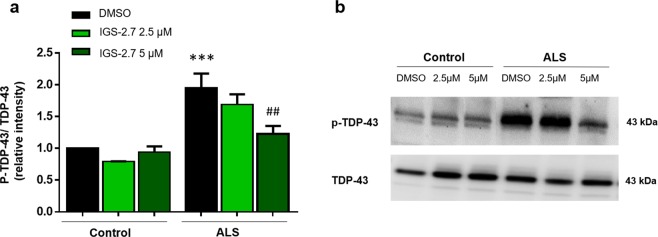
Effect of IGS-2.7 on TDP-43 phosphorylation in immortalized lymphocytes from control and sporadic ALS individuals. Immortalized lymphocytes were seeded at an initial density of 1 × 10^6^ × ml^−1^ in absence or presence of IGS-2.7 (2.5 and 5 μM). 24 h after drugs addition, cells were harvested and processed for Western blotting analysis. (**a**) Densitometric measurements were performed on individual immunoblots and values indicate the mean of p-TDP-43 versus the corresponding total TDP-43 levels ± SEM for experiments carried out with 4 different cell lines for each group. (**b**) Representative immunoblot is shown. Data were assessed by one-way ANOVA and *post hoc* Fisher’s analysis (***p < 0.001 significantly different from control cells; ^##^p < 0.01 and significantly different from untreated ALS cells).

Previous work from our laboratory demonstrated that lymphoblasts derived from FTD and ALS patients, easily available, could represent a suitable platform to search novel disease-modifying drugs^16,17^. For this reason, we decided to explore the biological profile of IGS-2.7 on lymphoblasts derived from sALS patients. The effect of IGS-2.7 in decreasing TDP-43 phosphorylation in control and sALS cells was assessed by Western blotting using a phospho-specific (S409/410) and anti-TDP-43 antibodies at two different compound concentrations. Figure 4 shows a representative immunoblot. Levels of phospho-TDP-43 were higher in sALS samples than in controls (healthy individuals), mimicking this pathological hallmark of ALS pathology. Treatment of lymphoblasts with 5 µM of IGS-2.7 for 24 h, resulted in a significant decrease in the levels of phosphorylation of TDP-43 in sALS lymphoblasts while no differences were observed in controls.

**Figure 5:**
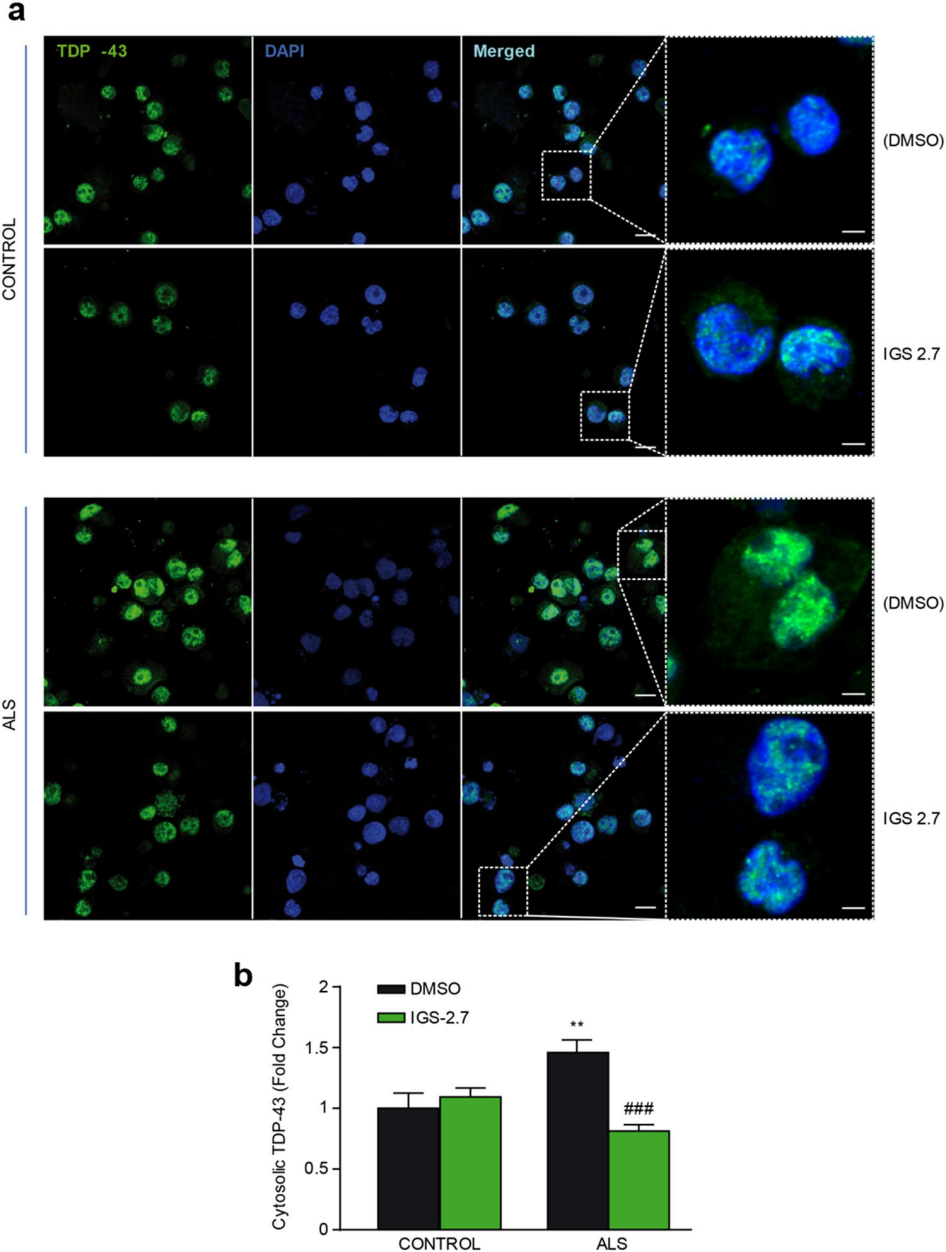
Subcellular localization of TDP-43 after IGS-2.7 treatment of lymphoblasts from control and ALS subjects. Lymphoblasts were seeded at 10^6^ cells × ml^−1^ and incubated in presence or absence of IGS-2.7 (5 μM) for 24 h. (**a**) Cells were stained with anti-TDP-43 antibody followed by a secondary antibody labeled with Alexa Fluor 488. DAPI was included in the mounting media to stain the nucleus. TDP-43 protein localization was assessed by confocal laser scanning microscopy. Merged images show that treatment with IGS-2.7 prevented the higher cytosolic accumulation (red arrows) of TDP-43 protein in ALS patients. Scale bars = 11 μM. Magnified cells from images are shown on the right panels for better visualization (Scale bars = 3 μm) (**b**) Quantification of TDP-43 cytosolic localization in lymphoblasts from ALS patients compared to controls. Data are expressed as mean ± SEM for experiments carried out with 4 different cell lines for each group. Data were assessed by one-way ANOVA and *post hoc* Fisher’s analysis (**p < 0.01 from control cells; ^###^p < 0.001 from untreated ALS cells).

Next, we addressed whether the treatment of IGS-2.7 on ALS lymphoblasts could normalize the mislocalization of TDP-43, thus recovering the TDP-43 homeostasis. Subcellular distribution of TDP-43 was assessed by immunostaining using a phospho-independent anti-TDP-43 antibody. Figure 5 shows how IGS-2.7 treatment prevented the cytosolic accumulation of TDP-43 in lymphoblasts from ALS patients with no effects on control cells.

### CK-1 expression in human spinal cord of control and sporadic ALS patients

**Figure 6:**
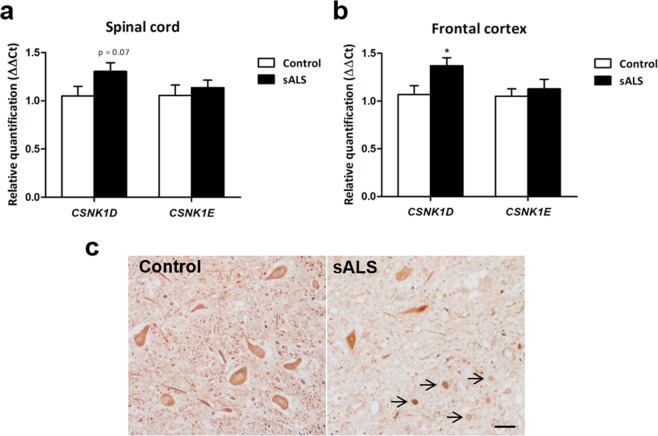
Relative expression of *CSNK1D* and *CSNK1E* mRNA levels in *post-mortem* samples from control and ALS subjects and immunohistochemical analysis. (**a**) *CSNK1D* and *CSNK1E* mRNA levels in the anterior horn of the spinal cord and (**b**) frontal cortex area 8 of sALS cases and controls, *p < 0.05. (**c**) CK-1δ immunoreactivity in the anterior horn of the spinal cord at lumbar level in control and ALS cases showing CK-1δ immunoreactivity in the cytoplasm of neurons and neuronal processes in control. Similar reaction is found in the remaining motor neurons in devastated anterior horn in the ALS case together with increased CK-1δ immunoreactivity in reactive glial cells (arrows). Paraffin sections, slightly counterstained with haematoxylin; scale bar = 25 µm.

To assess whether CK-1δ promotes TDP-43 phosphorylation in ALS patients, we analysed the expression levels of *CSNK1D* and *CSNK1E* genes and the kinase abundance of CK1 in spinal cord of control and sporadic ALS patients. Figure 6a shows the expression levels of *CSNK1D* and *CSNK1E* mRNA in the anterior horn of the spinal cord and frontal cortex area 8 in sALS cases. A tendency to increase of *CSNK1D* mRNA levels was observed in the anterior horn of spinal cord (p = 0.07) in sALS cases when compared with controls (Fig. 6a). Significant increase in *CSNK1D mRNA* levels occurred in frontal cortex of sALS cases when compared with controls (p = 0.025) (Fig. 6b). In contrast, no significant differences in *CSNK1E* mRNA levels were seen between control and sALS cases.

Immunohistochemistry for CK-1δ was performed thereafter in human spinal cord sections of 6 control and sporadic ALS cases. Figure 6C demonstrates diffuse CK-1δ immunoreactivity in the cytoplasm in both motor neurons from ALS and control cases with the exception of apparent increased immunoreactivity in shrunken neurons. Since the number of motor neurons was decreased in ALS cases, the amount of neuronal CK-1δ immunoreactivity was lower in ALS when compared with controls. However, increased CK-1δ immunoreactivity was observed in reactive glial cells in the anterior horn of ALS cases (Fig. 6C, right panel).

## Discussion

ALS is a fatal motor neurodegenerative disorder devoid of an effective therapy. The development of innovative drugs displaying neuroprotective and anti-inflammatory activities that might delay disease onset, slow its progression and/or prolong survival is a major therapeutic goal in ALS disorder. Deposition of intracellular aggregates of phosphorylated TDP-43 is a hallmark lesion in almost all cases of ALS, as well as in FTD^8^. Since TDP-43 protein plays an important role in RNA metabolism, apoptosis, cell division and plasticity^13,18–21^, it is likely that TDP-43 aggregation can promote neurodegeneration^22^. Therefore, strategies that prevent TDP-43 misfolding, aggregation and/or enhance clearance of pathological TDP-43 may serve as an important neuroprotective mechanism to counteract disease progression in ALS. With this purpose, we have explored here the therapeutic potential of inhibiting one of the key protein kinases involved in TDP-43 phosphorylation, protein kinase CK-1δ. We have tested the effectiveness of the brain permeable small molecule named as IGS-2.7, a potent inhibitor of CK-1δ with some residual inhibitory activity on CK-1ε, in an *in vivo* preclinical study using a TDP-43 transgenic mice model of ALS. It is important to remark that it is the first attempt to investigate a pharmacological treatment based on kinase inhibition in this mouse model of ALS. Moreover, we have extended our study to lymphoblasts from sporadic ALS patients, as we have shown the versatility of this human-cell based assay for drug discovery programs including FTD^23^.

Our results, shown that a chronic treatment with the compound IGS-2.7 effectively prevents the phosphorylation of TDP-43 *in vivo* in the spinal cord of TDP-43 transgenic mice, being this effect associated with an attenuation of most of the events that reflect the worsening of the pathological phenotype. This chronic treatment has no effect in the healthy animal (wild-type), despite the fact that CK1-δ is constitutively expressed. It seems that IGS-2.7 selectively prevents the aberrant hyperphosphorylation found in the transgenic TDP-43 mice. Whether the overactivation of CK-1δ in pathological conditions, is due to changes in mRNA, protein expression levels, posttranslational modifications, or in the sequestration of the enzyme in subcellular compartments^24^ remains to be elucidated. Among the pathological events ameliorated by CK-1δ inhibitor treatment, IGS-2.7 attenuated the typical animal weight losses, prevented the death of spinal motor neurons and reversed the glial reactivity affecting both microglial cells and especially astrocytes, which are particularly abundant in this experimental model. All these data strongly suggest that the inhibition of CK-1δ with the benzothiazole derivative IGS-2.7 may modulate TDP-43 toxicity *in vivo* by limiting TDP-43 phosphorylation, which situates this event in a key position to explain the benefits observed with this drug candidate in the preservation of spinal motor neurons. It is true that the recovery of these parameters is not complete but partial, which stresses the need to better optimize this treatment, either by elevating the active dose used or by combining IGS-2.7 with another neuroprotective agent (e.g riluzole). Furthermore, these rescued motor neurons should be involved in the muscular junction as choline acetyl transferase (ChAT) immunohistochemistry, an enzyme responsible for the synthesis of the neurotransmitter acetylcholine, is used in their quantification^25^.

Moreover, we used lymphoblastoid cell lines derived from sALS patients to mimic the central nervous system (CNS) dysfunction and investigated the effect of IGS-2.7 on TDP-43 pathology. Lymphoblasts from patients may reflect changes occurring in the CNS and represent a novel, easily accessible human-based cell model for drug screening and investigation^23^. Recently, we have shown that immortalized lymphocytes from sporadic ALS patients recapitulate pathological TDP-43 features^17^. Here, we report that treatment with the CK-1δ IGS-2.7 inhibitor improved TDP-43 homeostasis in this human cell-based sALS model. Benzothiazole IGS-2.7 not only decreases TDP-43 phosphorylation in cells derived from ALS patients but also corrects the subcellular localization of TDP-43, preventing the abnormal cytosolic TDP-43 accumulation in ALS lymphoblasts. This finding is in consonance with a previous work in which the effect of IGS-2.7 on the subcellular localization of TDP-43 was additionally evaluated in fractionated nuclear and cytoplasmic extracts from FTD-derived lymphoblasts^16^.

Finally, and in order to enhance the translational value of the results found in the *in vivo* model, we analysed the expression of CK-1δ and CK-1ε in the anterior horn of the spinal cord and frontal cortex area 8 in healthy subjects and sALS patients by quantitative RT-PCR. We have found a tendency to increase in the mRNA levels of *CSNK1D* in the anterior horn of the spinal cord and a significant increase in the frontal cortex area 8. In contrast, no differences in *CSNK1E* expression were found in the spinal cord and frontal cortex in sALS when compared with controls. Moreover, we analysed CK-1δ immunoreactivity in the anterior horn of spinal cord in control and ALS cases. CK-1δ expression was present in the cytoplasm of motor neurons of anterior horn without differences between control and ALS cases, with the exception of apparent increased immunoreactivity in shrunken neurons. However, we observed increased CK-1δ immunoreactivity in reactive glial cells in the anterior horn of ALS cases. This increase was not observed in control samples. These observations suggest the involvement of CK-1δ in human ALS pathology. The selective immunoreactivity of CK-1δ found in ALS reactive glial cells may also well correlate with the *in vivo* effect observed after the treatment with IGS-2.7 of reversion of the glial reactivity both in microglial cells and especially astrocytes.

In conclusion, our results show strong evidence to support that IGS-2.7 has neuroprotective properties, derived from its capability to reduce the phosphorylation of TDP-43. This has been seen *in vivo*, in TDP-43 mouse model, in which the inhibitor preserved motor neurons in ventral horn of spinal cord, and also *in vitro* using a human cell-based ALS model (lymphoblasts derived from sALS patients). In that model, IGS-2.7 treatment recovers TDP-43 homeostasis by decreasing its phosphorylation and enhancing the nuclear localization. Moreover, up-regulation of CK-1δ is shown in brain (frontal cortex) and spinal cord of sALS patients. Altogether, our results indicate that the inhibition of CK-1δ with selective inhibitors, specifically with the benzothiazole derivative IGS-2.7 used here, might represent a good therapeutic approach for disorders involved in TDP-43 protein alterations including ALS and FTD, among others. Only future clinical studies can confirm these results in patients. Moreover, it is worth mentioning that this CK-1δ inhibitor does not produce mutations, which makes it a good candidate for future pharmaceutical development^26^. Only future clinical trials will be able to confirm these results in patients.

## Methods

All reagents for cell culture assays were obtained from Invitrogen (Carlsbad, CA, USA). For Western blots (WB) experiments, PVDF (polyvinylidene difluoride) membranes were obtained from Bio-Rad (Richmond, CA, USA). Chemiluminescent reagent (ECL) for WB detection was purchased from Amersham (Uppsala, Sweden). All other reagents were of molecular grade. The *N*-benzothiazolyl-2-phenyl-acetamides derivative, CK-1δ inhibitor, IGS-2.7 was synthesized as previously described^27^ in our laboratory. Antibodies against human TDP-43 (10782-2-AP) and phospho (Ser409/410)-TDP-43 (22309-1AP) were purchased from Proteintech (Manchester, UK). Antibodies against α-tubulin (sc-23948), and GAPDH (sc-25778) were obtained from Santa Cruz Biotechnologies (Santa Cruz, CA, US), anti-GFAP antibody was obtained from DAKO (Glostrup, Denmark), anti-Iba-1 (019-19741) was purchased in Wako Chemicals (Richmond, VI, USA), and anti-ChAT antibody (PA5-29653) was obtained from Life Technologies (Carlsbad CA, US). Dilutions are shown in Table 1.

**Table 1:** Summary of antibodies used.

Antibody name	Company	Dilution
ChAT (PA5-29653)	Life Technologies	1:200
Iba-1 (019-19741)	Wako Chemicals	1:500
GFAP (Z0334)	DAKO	1:500
β-actin (sc-81178)	Santa Cruz	1:500
Human TDP-43 (10782-2-AP)	Proteintech	1:1000
Human ^Ser409/410^TDP-43 (22309-1AP)	Proteintech	1:500
α-tubulin (sc-23948)	Santa Cruz	1:1000
GAPDH (sc-25778)	Santa Cruz	1:500
Alexa Fluor 488 (R37118)	Life Technologies	1:200
CK-1δ	AbCam	1:100

### Animal procedures

Prp-hTDP-43(A315T) transgenic mice and non-transgenic littermate sibling were used as model in all the experiments. Mice were breeding in our animal facilities from initial breeders were purchased to Jackson Laboratories (Bar Harbor, ME, USA).

Offspring were genotyped for the TARDBP transgene which contain the A315T mutation following a procedure described previously^13^. Mice were housed in an environment with controlling temperature (22 ± 1 °C) and light (12-h light/dark cycle) with food and water available *ad libitum*. Experimental designs and procedures were approved by the ethical committees of the Complutense University and the regulatory institution (ref. PROEX 059/16) in accordance with the European Commission regulations (2010/63/EU) for the use of laboratory animals. Then, once genotyped, wild-type and transgenic mice were identified by numbered earmarks and, prior to the start of the different experiments, they were randomly allocated to the different treatment groups. For data collection, in all behavioral and histological analyses, researchers were blinded to the animal treatment, whereas for the biochemical analyses, due to the form in which the data are collected, blinding was not considered necessary, as previously described^28^.

### Lymphoblastic cell lines

Peripheral blood samples from 4 sALS patients and 4 age-matched control subjects were used for peripheral blood mononuclear cells (PBMCs) isolation on Lymphoprep™ density-gradient centrifugation according to the instructions of the manufacturer (Axis-Shield Po CAS, Oslo, Norway). Establishment of lymphoblastoid cell lines was performed in our laboratory by infecting peripheral blood lymphocytes with the Epstein–Barr virus as described previously^29^. All patients were diagnosed by applying the revised El Escorial criteria^30^ in the Hospital Doce de Octubre (Madrid, Spain). All procedures were approved by the Hospital Doce de Octubre and the Spanish Council of Higher Research Institutional Review Board and are in accordance with National and European Union Guidelines. Informed written consent was obtained from all the participants. Demographic and clinic characterization of individuals enrolled in this study can be found in Table 2.

**Table 2:** Demographic and clinical characterization of subjects included in this study.

	CONTROL	ALS
	(n = 4)	(n = 4)
Gender (M/F)	(1/3)	(1/3)
Family history	No	No
Age (y ± SD)	62 ± 9	68 ± 9
**Site of onset (n)**		
Bulbar	NA	4

M: male; F: female; y: years.

Lymphoblastoid cells lines were grown in suspension in T flasks in an upright position, in approximately 8 ml of RPMI-1640 medium that contained 2 mM L-glutamine, 100 μg/ml streptomycin/penicillin and 10% (v/v) fetal bovine serum and maintained in a humidified 5% CO_2_ incubator at 37 °C. Fluid was routinely changed every three days by removing the medium above the settled cells and replacing it with an equal volume of fresh medium as previously described^17^.

### Treatments and sampling

TDP-43 (A315T) transgenic male mice and wild-type mice were used to register disease progression and spinal cord decline in a pharmacological study using the CK-1δ inhibitor, IGS-2.7. This compound was synthetized in our laboratory following described procedures showing an IC_50_ value for CK-1δ of 23 nM and a residual IC50 of 840 nM for CK-1ε^27^. Mice (10 per group) were treated starting at the age of 65 days old, intraperitoneally, with IGS-2.7 (1 mg/kg of body weight) in 6.2% Tween 20 and 4% DMSO in saline buffer daily until sacrifice at the age of 90 days old. The rationale for this range of age has been explained in several studies^28,31^ based on the fast progression of this model compared to other TDP-43-based model^32^. Vehicle injections were administered to control animals. This dose was selected based on previous pharmacokinetic results of compound IGS-2.7, that shows good levels of the drug in plasma and brain after intraperitoneally and oral administration^16^. During all the treatment, we also recorded the physical appearance and the animal weight gain.

As we described previously^28^, after 25 days of chronic treatment, TDP-43(A315T) transgenic male mice and their corresponding wild-type animals were euthanized 24 h after the last injection of IGS-2.7 by decapitation and their spinal cords were dissected and rapidly removed. The spinal samples to be used for western blot analyses were frozen in 2-methylbutane cooled in dry ice and stored at −80 °C. The spinal samples to be used for histological analyses were fixed for one day at 4 °C in fresh 4% paraformaldehyde prepared in 0.1 M phosphate buffered-saline, pH 7.4. Samples were then cryoprotected by immersion in a 30% sucrose solution for a further day, and finally stored at −80 °C. Fixed spinal cords were sliced with a cryostat at the lumbar level (L4-L6) to obtain coronal sections (20 μm thick) that were collected on gelatine-coated slides. Sections were used for procedures of Nissl-staining and immunofluorescence.

### Histological procedures

#### Nissl staining

Sections of the spinal cord at the lumbar level (L4-L6) were used for Nissl staining following the protocol previously described^28^. We used cresyl violet 0.25% with 0.1% of acetic acid. Slices were dehydrated by an increased ethanol concentration series, sealed and cover-slipped with the non-aqueous mounting medium DPX (Sigma-Aldrich, San Luis, MI, USA). A Leica DMRB microscope (Leica, Wetzlar, Germany) and a Leica DFC300FX camera were used for slice observation and for taking photographs of the tissues. The number of total motor neurons in the ventral horn was counted in at least six slices to a minimum of 6 animals per experimental group. To count the number of stained motor neurons (>400 μm^2^) in the ventral horn, high resolutions photomicrographs were taken with a 10X objective under the same conditions of light, brightness and contrast. The final value for each group is the mean of all animals included in the study.

#### Immunofluorescence

Slices from TDP-43 (A315T) mice model and controls were used for immunofluorescence study. The protocol used was as described previously^28^. Tissue sections were pre-incubated for 1 h with Tris-buffered saline with 1% Triton X-100 (pH 7.5) for permeabilization. Then, sections were incubated overnight at 4 °C with a polyclonal anti-ChAT (1:200; Life Technologies, CA, USA), anti-Iba-1 (1:500; Wako Chemicals, Richmond, VI, USA) or anti GFAP (1:500; DAKO, Glostrup, Denmark) antibody. After several washes with Tris-buffered saline, tissues were incubated with an Alexa 488 secondary antibody conjugate (1:200; Life Technologies) for 2 h at 37 °C. For slice observation and photography, a Leica DMRB microscope and a Leica DFC300FX camera were used. Quantification of immunofluorescence was carried out on high-resolution photomicrographs that were taken with the 10X objective, under the same conditions of light, brightness and contrast. The software of analysis Image J (NIH; Bethesda, MD, USA) was used to quantify the mean density of labelling in a selective area. Six sections coming from at least six animals were analysed to establish the mean value for each group included in the study. The final data were normalized and were expressed relative to wild-type animals. Subcellular localization of TDP-43 was assessed in human lymphoblasts by immunofluorescence as previously described^17^. Cells (1 × 10^6^ ml^−1^) were incubated with and without IGS-2.7 (5 µM) for 24 h and then fixed for 30 min in 4% paraformaldehyde in PBS and blocked and permeabilized with 0.5% TritonX-100 in PBS-0.5% BSA for 60 min at room temperature. Cells were attached to poly-L-lysine coated coverslips using the Cytospin centrifuge at 700 rpm for 7 minutes before they were incubated overnight with the anti-TDP-43 polyclonal antibody. After removing the primary antibody, cells were washed with PBS and were incubated with Alexa Fluor 488-conjugated anti-rabbit antibody. Preparations were mounted on ProLong® Gold Antifade Reagent with DAPI (Thermo Fisher) allowing nuclear visualization. High-resolution images were acquired for ∼30 cells per group in n = 4 independent experiments using a confocal microscope Zeiss 510 equipped with a META detection system and a 63x oil immersion objective. Cytosolic TDP-43 levels per cell were quantified using the *Volocity* software (PerkinElmer, Waltham, MA, USA).

Tissue sections from 6 ALS cases and 6 age-controls were used for immunostaining study. Formalin-fixed, paraffin-embedded, de-waxed 4 µm-thick sections of human anterior horn of the spinal cord at the level of upper lumbar were boiled in citrate buffer for 20 min to retrieve protein antigenicity. Endogenous peroxidases were blocked by incubation in 10% methanol and 1% H_2_O_2_ for 15 min, followed by 3% normal horse serum incubation and washed in distilled water. The sections were incubated at 4 °C overnight with the mouse monoclonal anti-CK-1δ antibody (Abcam, Cambridge, UK) used at a dilution of 1:100. Following incubation with the primary antibody, the sections were incubated with EnVision+ system peroxidase (Dako, Agilent Technologies, Santa Clara, CA, USA) for 30 min at room temperature. The peroxidase reaction was visualized with diaminobenzidine and H_2_O_2_. Control of the immunostaining included omission of the primary antibody and no signal was obtained following incubation with only the secondary antibody. Finally, the sections were slightly counterstained with haematoxylin.

### Immunoblotting analysis

Samples of spinal cords from TDP-43 (A315T) mice and controls were used to assess TDP-43 phosphorylation state as previously described^33^.

Mouse spinal cords were lysed in RIPA buffer supplemented with a protease and phosphatase inhibitor cocktail (Roche, Mannhein, Germany). To prepare whole-cell extract, cells were harvested, washed in PBS and then lysed in ice-cold lysis buffer as described. The protein content of the extracts was determined by the Pierce BCA Protein Assay kit (Thermo Scientific). 20–50 μg of protein were fractionated on SDS poly-acrylamide gel and transferred to poly-vinylidene fluoride (PVDF) membranes (Millipore, Billerica, MA, USA). The membranes were then blocked with 5% bovine serum albumin (BSA) (Sigma) for 1 h and then incubated overnight at 4 °C, with primary antibodies at the dilutions indicated in Table 1. Signals from the primary antibodies were amplified using species-specific antisera conjugated with horseradish peroxidase (Bio-Rad) and detected with a chemiluminescent substrate detection system ECL. Protein band densities were quantified using a ChemiDoc station with Quantity One 1D analysis software (Bio-Rad Laboratories, Madrid, Spain). All the immunoblots can be found in the supplementary data file.

### Human cases

*Post-mortem* fresh-frozen lumbar spinal cord (SC) and frontal cortex (FC) (Brodmann area 8) samples were obtained from the Institute of Neuropathology HUB-ICO-IDIBELL Biobank following the guidelines of Spanish legislation on this matter (Real Decreto 1716/2011) and approval of the local ethics committee of the Bellvitge University Hospital-IDIBELL. The *post mortem* delay varied from 2 hours and 15 minutes to 17 hours. One hemisphere was immediately cut in coronal sections, and selected areas of the encephalon were rapidly dissected, frozen on metal plates over dry ice, placed in individual labeled air-tight plastic bags, and stored at −80 °C until use for biochemical studies. The other hemisphere was fixed by immersion in 4% buffered formalin for no less than 3 weeks for morphological studies. The lumbar anterior spinal cord was dissected on a dry-ice frozen plate under a binocular microscope at a magnification x4. Cases with concomitant pathologies were not considered in the present study. The series included 18 sALS cases and 30 controls. The anterior horn of the spinal cord was available in 14 sALS (mean age 57 years; 6 men and 8 women) and the frontal cortex area 8 in 15 sALS (mean age 54 years; 11 men and 4 women). The spinal cord and frontal cortex were available in 11 cases^34^. Mutations related to ALS and/or FTD, including *SOD1, C9ORF72, TARDBP* and *FUS*, were excluded in every case. Age-matched control cases had not suffered from neurologic and psychiatric disorders and did not show alterations^34^.

### RNA extraction and RT-qPCR validation

RNA from frozen spinal cord and frontal cortex area 8 was extracted following the instructions of the supplier (RNeasy Mini Kit, Qiagen® GmbH, Hilden, Germany). RNA integrity and 28S/18S ratios were determined with the Agilent Bioanalyzer (Agilent Technologies Inc, Santa Clara, CA, USA) to assess RNA quality, and the RNA concentration was evaluated using a NanoDrop™ Spectrophotometer (Thermo Fisher Scientific). Complementary DNA (cDNA) preparation used High-Capacity cDNA Reverse Transcription kit (Applied Biosystems, Foster City, CA, USA) following the protocol provided by the supplier. Parallel reactions for each RNA sample run in the absence of MultiScribe Reverse Transcriptase to assess the lack of contamination of genomic DNA. TaqMan RT-qPCR assays were performed in duplicate for each gene on cDNA samples in 384-well optical plates using an ABI Prism 7900 Sequence Detection system (Applied Biosystems, Life Technologies, Waltham, MA, USA). For each 10 μL TaqMan reaction, 2.25 μL cDNA was mixed with 0.25 μL 20x TaqMan Gene Expression Assays and 2.50 μL of 2x TaqMan Universal PCR Master Mix (Applied Biosystems). Relative expression of casein kinase 1 isoform delta (*CSNK1D*) and epsilon (*CSNK1E*) genes was assessed using the following TaqMan probes: Hs01095996_m1 and Hs01017895_m1, respectively. The values of glucuronidase Beta (*GUS-β*) gene (Hs00939627_m1) were used as internal controls for normalization of frontal cortex samples^34^ and Hypoxanthine Phosphoribosyl transferase 1 (*HPRT-1*), (Hs02800695_m1) values were used as internal controls^34^ for normalization of spinal cord samples. The parameters of the reactions were 50 °C for 2 min, 95 °C for 10 min, and 40 cycles of 95 °C for 15 sec and 60 °C for 1 min. Finally, capture of all TaqMan PCR data used the Sequence Detection Software (SDS version 2.2.2, Applied Biosystems). The double-delta cycle threshold (ΔΔCT) method was used to analyse the data; and statistical study was performed using T-student test. The significance level was set at *p < 0.05, **p < 0.01 and ***p < 0.001 vs. control group^34^.

### Statistical analysis

Data are expressed as mean values ± SEM and statistical analysis was performed using the Graph Pad software (version 6.0). Significant differences between the groups were evaluated using two-way ANOVA test followed by a Bonferroni *post hoc* comparison. A p-value lower than 0.05 was used as the limit for statistical significance. The sample sizes used in the different experimental groups of all experiments were always >5. One-way ANOVA was used to investigate differences between lymphoblasts incubated in the absence or presence of IGS2.7.

## Supplementary information

Supplemmentary figures.

## Data Availability

The datasets generated during and/or analysed during the current study are available from the corresponding author on reasonable request.
