# Supplementary material for: Motor neuron preservation and decrease of in vivo TDP-43 phosphorylation by protein CK-1δ kinase inhibitor treatment
Source: Sci Rep. 2020 Mar 10;10:4449. doi: 10.1038/s41598-020-61265-y (PMC7064575; doi:10.1038/s41598-020-61265-y)

# Motor neuron preservation and decrease of *in vivo* TDP-43 phosphorylation by protein CK-1δ kinase inhibitor treatment

Loreto Martinez-Gonzalez,[1] Carmen Rodríguez-Cueto,[2-4] Diego Cabezudo,[2] Fernando Bartolome,[4,5] Pol Andrés-Benito,[4,6,7] Isidro Ferrer,[4,6,7] Carmen Gil,[1,4] Ángeles Martin-Requero,[1,4] Javier Fernández-Ruiz,[2-4] Ana Martinez,[1,4] * and Eva de Lago[2-4].*

[1]Centro de Investigaciones Biológicas-CSIC, Ramiro de Maeztu 9, 28040 Madrid, (Spain).

[2]Instituto Universitario de Investigación en Neuroquímica, Departamento de Bioquímica y Biología Molecular, Facultad de Medicina, Universidad Complutense, Madrid (Spain).

[3]Instituto Ramón y Cajal de Investigación Sanitaria (IRYCIS), Madrid (Spain).

[4]Centro de Investigación Biomédica en Red de Enfermedades Neurodegenerativas (CIBERNED), Madrid (Spain).

[5]Instituto de Investigación Sanitaria Hospital 12 de Octubre (imas12), 28041, Madrid (Spain)

[6]Department of Pathology and Experimental Therapeutics, University of Barcelona, Hospitalet de Llobregat, (Spain)

[7]Bellvitge University Hospital, IDIBELL (Bellvitge Biomedical Research Centre), Hospitalet de Llobregat, (Spain).

***** ana.martinez@csic.es
* elagofem@ucm.es

Supplemmentary figure 1

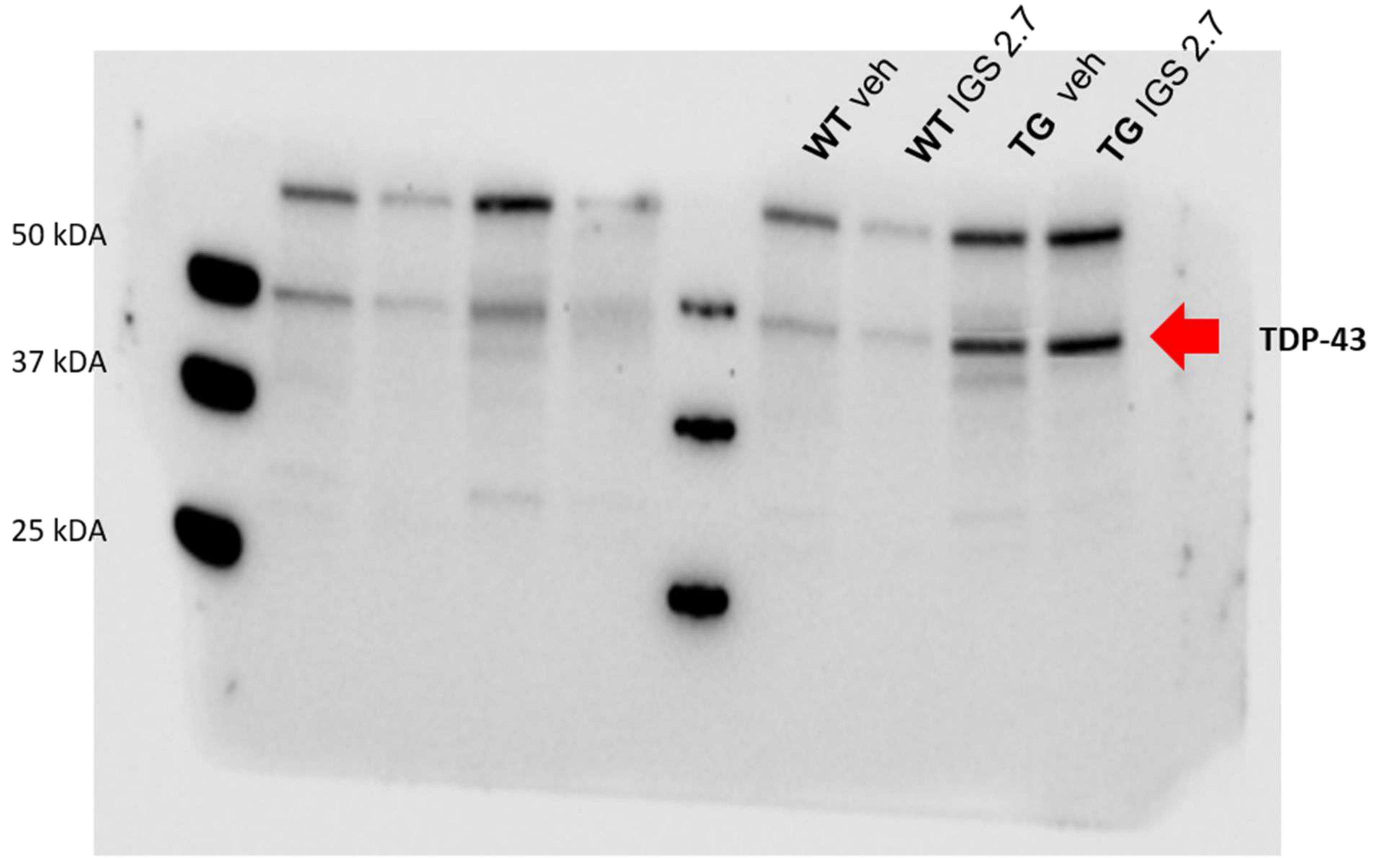


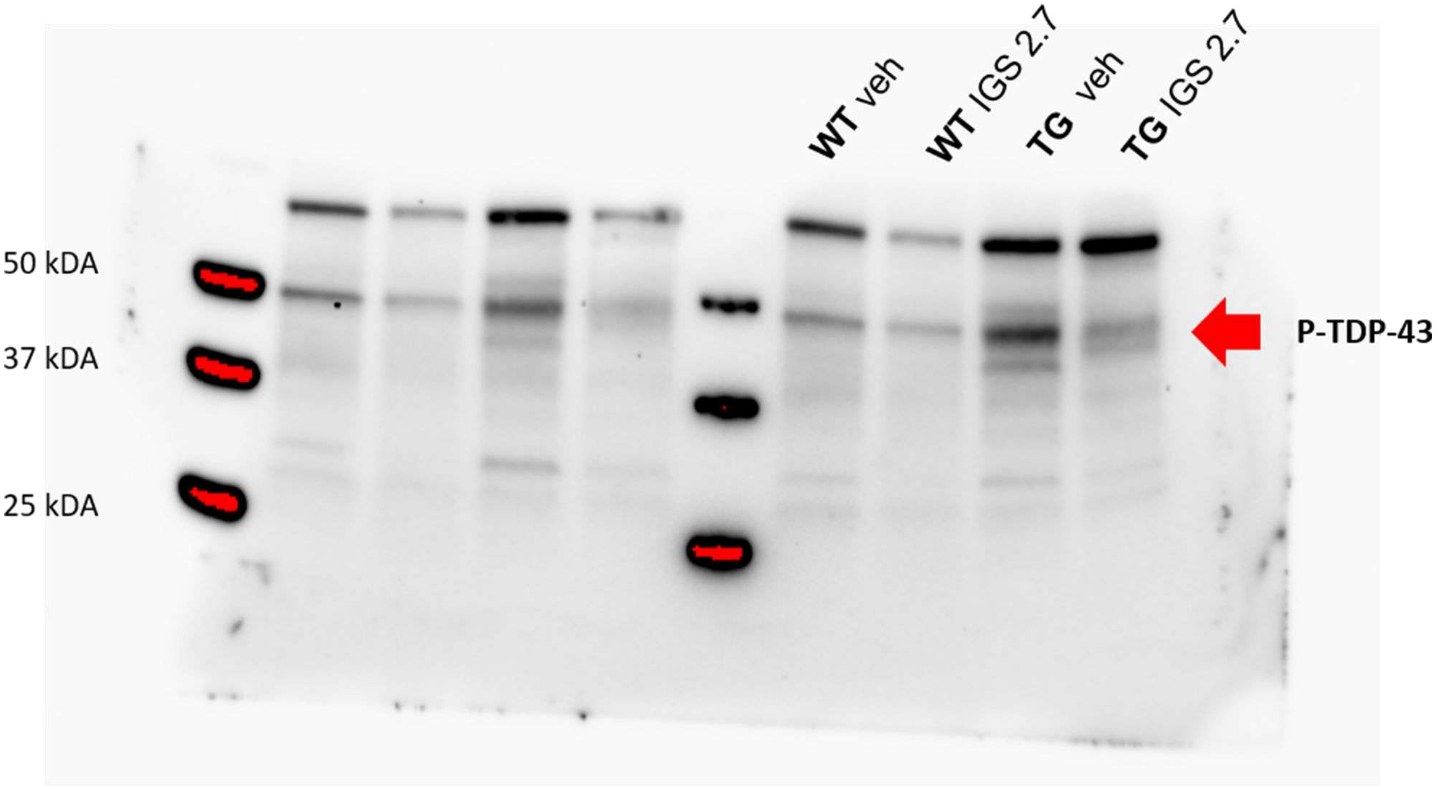

Supplemmentary figure 2

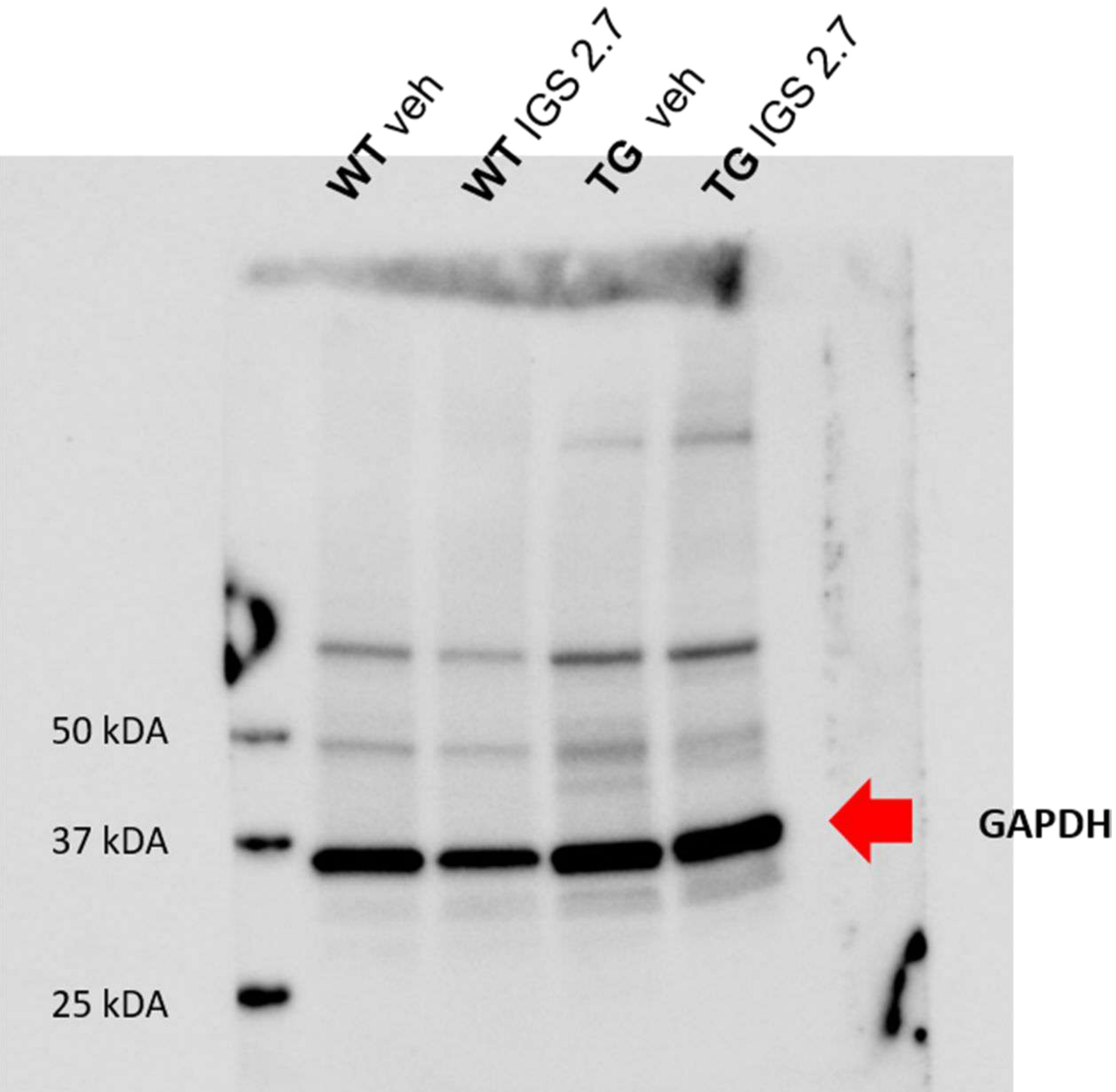

Supplemmentary figure 3

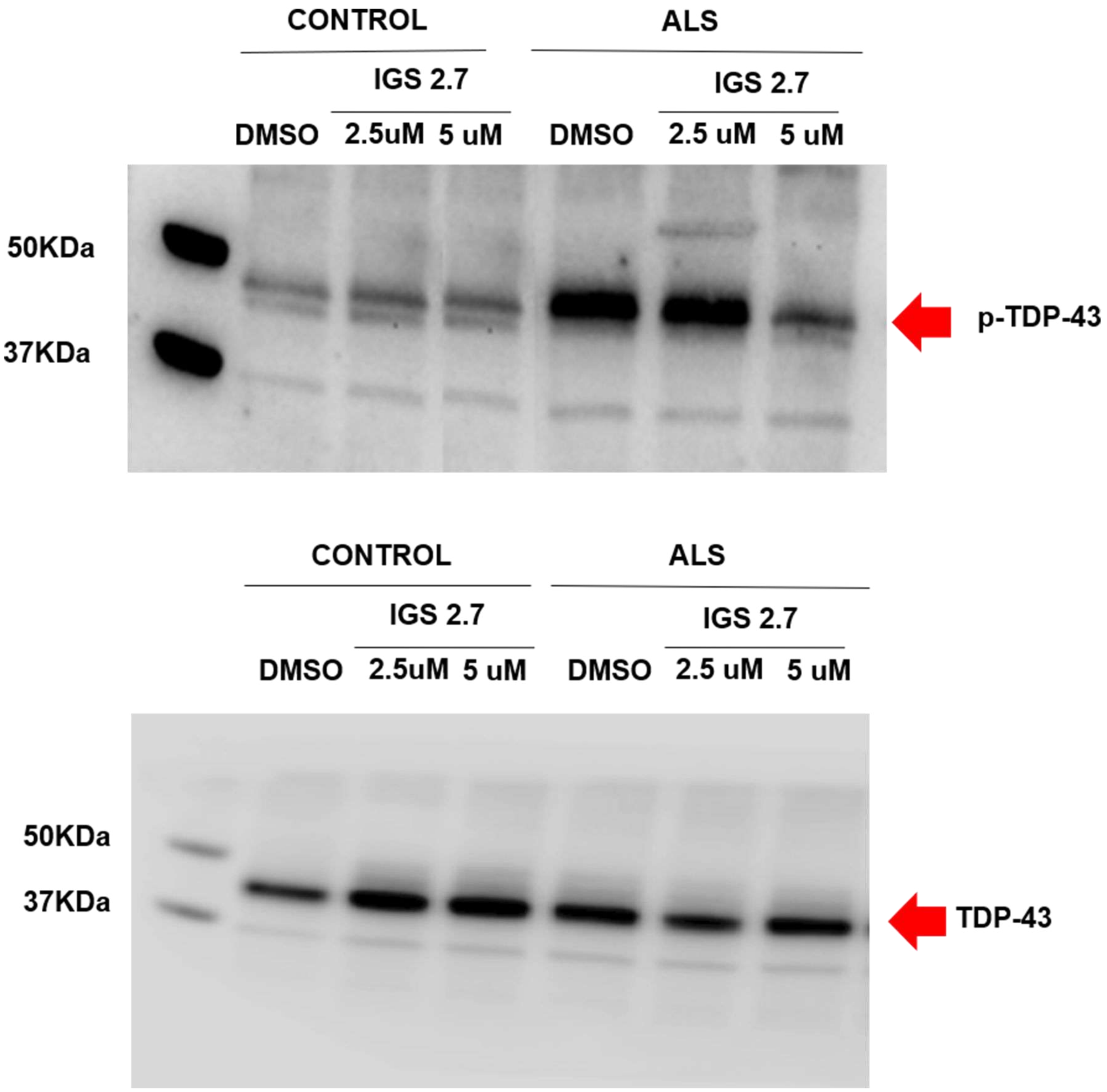

Supplement: Supplementary file 1 — Supplemmentary figures. [file 41598_2020_61265_MOESM1_ESM.pdf]
